# Danhong Injection Attenuates Cerebral Ischemia-Reperfusion Injury in Rats Through the Suppression of the Neuroinflammation

**DOI:** 10.3389/fphar.2021.561237

**Published:** 2021-04-13

**Authors:** Haixia Du, Yu He, Yuanjiang Pan, Mengdi Zhao, Zhiwei Li, Yu Wang, Jiehong Yang, Haitong Wan

**Affiliations:** ^1^ College of Basic Medical Science, Zhejiang Chinese Medical University, Hangzhou, China; ^2^ College of Pharmaceutical Science, Zhejiang Chinese Medical University, Hangzhou, China; ^3^ Department of Chemistry, Zhejiang University, Hangzhou, China; ^4^ College of Life Science, Zhejiang Chinese Medical University, Hangzhou, China

**Keywords:** Danhong injection, cerebral ischemia-reperfusion injury, neuroinflammation, central nervous system, NF-κB and MAPK signaling pathways

## Abstract

Neuroinflammation is one of the major causes of damage of the central nervous system (CNS) and plays a vital role in the pathogenesis of cerebral ischemia, which can result in long-term disability and neuronal death. Danhong injection (DHI), a traditional Chinese medicine injection, has been applied to the clinical treatment of cerebral stoke for many years. In this study, we investigated the protective effects of DHI on cerebral ischemia-reperfusion injury (CIRI) in rats and explored its potential anti-neuroinflammatory properties. CIRI in adult male SD rats was induced by middle cerebral artery occlusion (MCAO) for 1 h and reperfusion for 24 h. Results showed that DHI (0.5, 1, and 2 ml/kg) dose-dependently improved the neurological deficits and alleviated cerebral infarct volume and histopathological damage of the cerebral cortex caused by CIRI. Moreover, DHI (0.5, 1, and 2 ml/kg) inhibited the mRNA expressions of tumor necrosis factor-α (TNF-α), interleukin-1β (IL-1β), intercellular cell adhesion molecule-1 (ICAM-1), cyclooxygenase-2 (COX-2), and inducible nitric oxide synthase (iNOS) in ischemic brains, downregulated TNF-α, IL-1β, and monocyte chemotactic protein-1 (MCP-1) levels in serum, and reduced the neutrophil infiltration (myeloperoxidase, MPO) in ischemic brains, in a dose-dependent manner. Immunohistochemical staining results also revealed that DHI dose-dependently diminished the protein expressions of ICAM-1 and COX-2, and suppressed the activation of microglia (ionized calcium-binding adapter molecule 1, Iba-1) and astrocyte (glial fibrillary acidic protein, GFAP) in the cerebral cortex. Western blot analysis showed that DHI significantly downregulated the phosphorylation levels of the proteins in nuclear factor κB (NF-κB) and mitogen-activated protein kinas (MAPK) signaling pathways in ischemic brains. These results indicate that DHI exerts anti-neuroinflammatory effects against CIRI, which contribute to the amelioration of CNS damage.

## Introduction

Cerebral ischemia, also known as stroke, is a clinical common and refractory cerebrovascular disease that seriously harms human health. There are approximately 15 million new stroke patients worldwide each year, resulting in death of about 6 million people from the disease and making it a major cause of disability and death (Moskowitz et al., 2010; Eltzschig and Eckle, 2011). Ischemic stroke is the most common among the main pathological subtypes of stroke, accounting for approximately 85% (Kim and Roh, 2016). As a medical emergency, timely recovery of blood flow and concomitant reoxygenation in the ischemic zone to reduce the damage caused by ischemic stroke are still the only internationally recognized treatment method (Zhu et al., 2018). However, the subsequent reperfusion after the restoration of blood supply can also aggravate further damage of brain tissues, resulting in more severe neurological damage and brain dysfunction, called cerebral ischemia-reperfusion injury (CIRI). To date, recombinant tissue plasminogen activator (t-PA) is the only therapy approved by the Food and Drug Administration (FDA) in the United States for the treatment of acute ischemic stroke, but its drawback is that its application is limited by the narrow therapeutic window (Sahota and Savitz, 2011). In addition, thrombolytic therapy with t-PA infusion also has potential complications including neurotoxicity, hemorrhagic transformation, and poor thrombolytic perfusion rate, so less than 5% of patients with ischemic stroke can benefit from t-PA therapy (Mozaffarian et al., 2016; Chen et al., 2018). Thus, there is an urgent need to develop additional effective drugs for the treatment of CIRI.

The CIRI involves in a wide range of neuropathic alteration and a series of pathophysiological processes, mainly including inflammatory pathways, energy metabolism disorders, apoptosis, free radical-induced neuronal damage, and many other factors (Sahota and Savitz, 2011; Chamorro et al., 2016; Xie et al., 2018). Although the mechanisms of CIRI are complex, there are accumulating evidences that neuroinflammation plays a vital role in the pathogenesis and treatment of cerebral ischemic stroke (Eltzschig and Eckle, 2011; Jiang et al., 2014). In the early stage of inflammation, during cerebral ischemia, microglia (ionized calcium-binding adapter molecule 1, Iba-1) and astrocyte (glial fibrillary acidic protein, GFAP) are activated and adhesion molecules (such as intracellular adhesion molecule-1, ICAM-1) are located on cell surface to assist cells sticking to the vascular endothelium; then, chemokines and cytokines cross the wall of activated blood vessels to reach the ischemic penumbra (Tuttolomondo et al., 2011; Wang Y. et al., 2013; Li et al., 2016). In the later stage of inflammation, there are a lot of inflammatory substances in the ischemic penumbra, especially tumor necrosis factor (TNF)-α, interleukin (IL)-1β, and IL-6 (Cheng et al., 2019). Nuclear factor kappa B (NF-κB) is an important regulator of many downstream inflammatory factors, including inducible nitric oxide synthase (iNOS), TNF-α, and cyclooxygenase (COX)-2, and thus participates in a variety of cellular functions, such as inflammation, cell proliferation, and apoptosis (Zhang et al., 2013; van Delft et al., 2015). These key factors of the NF-κB signaling pathways can trigger neuroinflammatory responses and result in CIRI. Moreover, the mitogen-activated protein kinases (MAPK) signaling pathway has been reported to be related to the cell proliferation, differentiation, senescence, and apoptosis (Sun Y. et al., 2015). The MAPK signaling pathway has also been involved in the regulation of inflammatory cytokines and associated with the pathogenesis of the CIRI, and this pathway might represent a novel therapeutic target for neuroprotection (Che et al., 2017). Accordingly, studies that inhibit the activation of key regulatory factors in the NF-κB and MAPK signaling pathways may provide a basis for discovering novel therapeutic targets for stroke patients. Therefore, the corresponding anti-neuroinflammatory therapy agent has a good application prospect in alleviating CIRI.

Traditional Chinese medicine (TCM) has a history of more than 2,000 years and might be used as a supplement and alternative method to treat and prevent cardiovascular disease (Hao et al., 2017). Meanwhile, some TCM medications have gained widespread clinical applications and also show effective therapeutic effects in preventing and treating CIRI (Sun K. et al., 2015; Han et al., 2017). Danhong injection (DHI), as one of the most popular TCM medications for the treatment of cardiovascular and cerebrovascular diseases, is a standardized product extracted from *Salvia miltiorrhiza* Bunge and *Carthamus tinctorius* L., and the dose ratio of raw material is 3:1 (Wan et al., 2018; Feng et al., 2019). The major representative components in DHI are danshensu, salvianic acid, 3, 4-dihydroxybenzaldehyde, protocatechuic aldehyde, and rosmarinic acid; these compounds are related to pharmacological effects of DHI (Li et al., 2019; Xu et al., 2019). DHI has been used extensively in the clinical treatment of coronary heart disease and acutely cerebral infarction for many years (He et al., 2012). More experimental evidences show that DHI exerts diverse pharmacological properties by modulating multiple molecular targets (Feng et al., 2019), including recovery of cerebral glucose metabolism by serial ^18^F-labeled fluoro-2-deoxyglucose (^18^F-FDG)-micro positron emission tomography (PET) imaging and enhancing neurogenesis (Wang et al., 2014), angiogenesis-promoting actions by up-regulating expressions of the related factors of angiogenesis (Wan et al., 2018), and antiapoptotic by activating phosphatidylinositol 3-kinase-protein kinase B (PI3K-Akt) signaling pathway (Feng et al., 2020). Clinically, a network meta-analysis of randomized controlled trials found that DHI combined with other regimens had the highest effective rate in the treatment of acute cerebral infarction (Liu et al., 2018). Recent, studies have demonstrated that DHI exerts anticardiac hypertrophic effects and anti-inflammatory activities on LPS-induced endothelial inflammation by regulating the p38 and NF-κB signaling pathways (Mao et al., 2016; Lyu et al., 2017). Therefore, it is a response to assume that DHI might be an underlying anti-inflammatory therapy agent for CNS injury. In the present study, we investigated the protective effects of DHI against CIRI in rats and explored the potential anti-neuroinflammatory properties involved in the NF-κB and MAPK signaling pathways.

## Materials and Methods

### Reagents

TTC (2, 3, 5-triphenyltetrazolium chloride) was obtained from Sigma-Aldrich (Saint Louis, MO, United States). The enzyme-linked immunosorbent assay (ELISA) kits for TNF-α (Batch no. CK-E30635R), IL-1β (Batch no. CK-E30419R), and monocyte chemoattractant protein-1 (MCP-1, Batch no. CK-E30495R) were purchased from Yuanye Biotech Co., Ltd. (Shanghai, China), and myeloperoxidase (MPO, Batch no. A044) was obtained from Jiancheng Bioengineering Institute (Nanjing, China). For immunohistochemistry assays, primary antibodies against ICAM-1 (Batch no. ab206398), Iba-1 (Batch no. ab178847), and GFAP (Batch no. ab49874) were purchased from Abcam (Cambridge, MA, United States), and COX-2 (Batch no. #12282) was purchased from Cell Signaling Technology (Danvers, MA, United States). For western blot assays, antibodies of NF-κB p65 (Batch no. #8242), phospho-NF-κB p65 (p-NF-κB p65, Batch no. #3033), inhibitor of NF-κBα (IκBα, Batch no. #4814), p-IκBα (Batch no. #2859), c-Jun-N-terminal kinase (JNK, Batch no. #9252), and p-JNK (Batch no. #4671), and extracellular signal-related kinases (ERK1/2, Batch no. #4695), p-ERK1/2 (Batch no. #4370), P38 (Batch no. #8690), and p-P38 (Batch no. #4511), were obtained from Cell Signaling Technology (Danvers, MA, United States), and β-Actin (Batch no. MAB8929) antibody was purchased from Santa Cruz Biotech Co., Ltd. (California, United States).

### Quality Control of Danhong Injection

DHI was kindly provided by Shandong Buchang Pharmaceutical Co., Ltd. (Shandong, China, Batch no. 18011035), with a permission number (Z20026866) approved by China’ State Food and Drug Administration. In accordance with the corresponding quality control standard, the quality control of DHI was performed by high-performance liquid chromatography (HPLC) analysis with an established method in our laboratory. The major constituents and their relative content in DHI are identified, containing danshensu (1350 µg/ml), protocatechualdehyde (312 µg/ml), rosmarinic acid (246 µg/ml), caffeic acid (5.5 µg/ml), and salvianolic acid A (4420 µg/ml) and salvianolic acid B (396 µg/ml), respectively. Chromatogram of DHI, chemical structures, and relative content of major compounds are shown in Supplementary Figure S1.

### Animals

Adult male Sprague-Dawley (SD) rats (280 ± 20 g) were purchased from the Animal Center of Zhejiang Chinese Medical University (Hangzhou, China). Rats were maintained under controlled temperature (20 ± 2°C) and humidity (50 ± 10%). Animals were housed in a standard animal laboratory room with 12 h light/dark cycle and were allowed free access to food and water. All experiments were authorized by the Institutional Animal Care and Use Committee of Zhejiang Chinese Medical University [SCXK (Zhe) 2013–0184]. All animals during the experiment were cared in compliance with the Guide for the Care and Use of Laboratory Animals published by the United States National Institutes of Health (NIH Publications, No. 80–23, revised in 1996).

### Animal Model of Cerebral Ischemia-Reperfusion Injury

All rats were adaptively fed for 1 week and kept fasting without water for 12 h prior to surgery. Middle cerebral artery occlusion and reperfusion (MCAO/R) was induced based on the modified Zea Longa’s method (Longa et al., 1989). All rats were anesthetized with 3% pentobarbital sodium (2 ml/kg, i. p.). After a midline skin incision in the neck, the right common carotid artery (CCA), external carotid artery (ECA), and internal carotid artery (ICA) were exposed, respectively. The CCA and ECA were ligated, and the rounded tip was incised to place a poly-l-lysine-coated 4–0 nylon suture (Sunbio Biotech Co., Ltd. Beijing, China). The nylon suture was inserted about 18 ± 2 mm from right ICA to the middle cerebral artery. After 60 min of MCAO, the nylon filament was removed to restore the blood flow and permit reperfusion. The same procedures were performed on rats without the insertion of the nylon monofilament suture in the sham group. During the whole experiment, the body temperature of all rats should be maintained at 37°C.

### Groups and Drug Delivery

Rats were randomly separated into five groups: sham group, model group (MCAO), and DHI-treated groups (0.5 ml/kg, 1 ml/kg, and 2 ml/kg), respectively. The drug administration dose or period was determined based on the preliminary experiment results of the research group and the characteristics of the CIRI model. The equivalent dose of DHI for the adult of 60 kg weight was 10 ml per day, which was converted into the medium dose of rats (1 ml/kg). Based on the medium dose of DHI, the low and high doses were set. The rats in the DHI group were administrated *via* caudal vein immediately and 6 h after reperfusion, respectively, while those in the sham and model groups were given the same amount of physiological saline.

### Neurological Deficit Scores and Body Weight Examination

About 24 h after reperfusion, neurological deficit score (NDS) was measured after CIRI according to Zea Longa’s scoring criteria (Longa et al., 1989) as follows: 0 point, no neurological deficit scored; mild neurological deficit (with contralateral forepaw flexion and adduction) scored 1; moderate neurological deficit (turning to the contralateral side) scored 2; severe neurological deficit (slumping to the contralateral side when standing) scored 3; no autonomous activity with consciousness loss or unable to revive scored 4. In general, rats with higher score indicated more severe neurological deficits. In this study, only the rats with scores 1–3 after CIRI were selected as successful models.

Moreover, the rats before and after MCAO were weighed to evaluate the weight loss percentage.

### Infarct Volume Examination

About 24 h after reperfusion, rats were deeply anesthetized with 3% pentobarbital sodium (2 ml/kg, i. p.) and sacrificed by rapid decapitation. Brain tissues were collected and frozen at −20°C for 15 min and then sectioned into 2 mm thick coronal sections. These sections were immediately stained in 2% TTC solution at 37°C for 15 min under dark conditions, and the staining results were photographed and analyzed. Normal brain tissue was stained red, while the infarct brain tissue remained unstained. Image-ProPlus 6.0 software (Media Cybernetics, Warrendale, United States) was used to evaluate and quantify the infarction volume. The total infarct volume was calculated as the summation of the infarct area in six brain slices and expressed as a percentage of the total volume of slices (Cui et al., 2017). The formula is as follows: Infarct volume = (infarct volume of slices/total volume of slices) × 100%

### Histopathological Analysis

The rats were perfused first with ice-cold physiological saline and then fixed with frozen 4% paraformaldehyde at 4°C. The brain tissues were removed and fixed with 4% paraformaldehyde. Then, they were dehydrated in an ethanol series and embedded in paraffin according to the standard protocols. Finally, the coronal sections (3–4 μm) were stained with hematoxylin and eosin (H&E), and the hispathological change in the penumbra of ischemic ipsilateral parietal cortex area (see the schematic diagram at Supplementary Figure S2) was observed through a light microscope (Olympus, Tokyo, Japan) at a magnification of ×100 and ×200.

### Real-Time Polymerase Chain Reaction Analysis

Total RNA was isolated from ischemic brain tissues using the TRIzol reagent (Carlsbad CA92008, United States) according to the instructions provided by the manufacturer. And, the concentration of RNA was detected by ultraviolet visible spectrophotometer (Beckman, CA, United States). Then, the cDNA was obtained by reverse-transcription reaction at 25°C for 10 min, 42°C for 15 min, and 85°C for 5 min. Real-time PCR analysis was prepared in a reaction mixture consisting of Power SYBR® Green PCR Master Mix. The amplification protocol was as follows: denaturation at 95°C for 3 min, followed by 40 cycles at 95°C for 15 s, 53°C for 30 s, and 72°C for 15 s. The RT-PCR data were analyzed using the ABI 7500 sequence detection system (Applied Biosystems Co., California, CA). The primers for TNF-α, IL-1β, ICAM-1, COX-2, iNOS, and glyceraldehyde 3-phosphate dehydrogenase (GAPDH) (Sangon Biotech, Shanghai, China) are shown in Table 1. Using GAPDH as an internal reference, the relative mRNA expressions of target genes were calculated using the 2^−ΔΔCt^ formula.

**TABLE 1:** Primer sequences used for RT-PCR analysis.

Gene	Product size (bp)	Forward (5′-3′)	Reverse (5′-3′)
TNF-α	197	ACA​AGG​AGG​AGA​AGT​TCC​CAA​AT	GTC​TTT​GAG​ATC​CAT​GCC​ATT​G
IL-1β	131	AAT​ACC​ACT​TGT​TGG​CTT​A	TGT​GAT​GTT​CCC​ATT​AGA​C
ICAM-1	184	AAACGGGAGATGAATGGT	TCT​GGC​GGT​AAT​AGG​TGT​A
COX-2	376	ACGGACTTGCTCACTTTG	GGA​GAA​CAG​ATG​GGA​TTA​C
iNOS	700	GCA​TCC​CAA​GTA​CGA​GTG​GT	GAA​GGC​GTA​GCT​GAA​CAA​GG
GAPDH	161	GTC​GGT​GTG​AAC​GGA​TTT​GG	GTGCCGTTGAACTTGCCG

### Measurement of Inflammatory Cytokines in Serums and Brain Tissues

About 24 h after reperfusion, blood samples were collected from the rat hearts and centrifuged at 1466 × *g* for 15 min at 4°C. Then, the serum samples were assessed for inflammatory cytokine (TNF-α, IL-1β, and MCP-1) levels using the corresponding ELISA kits according to the manufacturer’s instructions. The values of optical density (OD) were measured at 450 nm.

The activity of MPO was evaluated by quantitative measurement of neutrophil infiltration. About 24 h after reperfusion, the brain tissues were quickly removed and collected. MPO activity was measured with an assay kit according to the instruction of the manufacturer; the results are expressed as U/g of tissue.

### Immunohistochemical Analysis

The rat brain tissues of each group were fixed in 4% paraformaldehyde at 4°C for 24 h. Paraffin-embedded were rehydrated, washed in PBS, and rinsed with 3% H_2_O_2_ for 15 min to block endogenous peroxidase. Next, the sections were blocked with 10% normal goat serum at 37°C for 20 min and incubated with the antibodies against ICAM-1 (1:1800), COX-2 (1:600), Iba-1 (1:2500), and GFAP (1:400) overnight at 4°C. The next morning, the sections were washed three times in PBS and then treated with secondary antibodies for 40 min at 37°C. Subsequently, the sections were washed three times with PBS and then colored with 3, 3′-diaminobenzidine. The images were observed using a light microscope. Five visual fields (100× and 200× magnification) in the penumbra of ischemic ipsilateral parietal cortex area (see the schematic diagram at Supplementary Figure S2) were selected and photographed with a fluorescence microscope (Olympus BX60, Japan). Furthermore, the analyses of the results were calculated with Image-ProPlus 6.0 software (Media Cybernetics, Warrendale, United States).

### Western Blot Analysis

About 24 h after reperfusion, the ischemic brain tissues were isolated and total protein was extracted using a tissue protein extraction kit (Beyotime Biotech Co. Ltd., Beijing, China), according to the manufacturer's protocol. The 100 µg of total protein samples were loaded onto the sodium dodecyl sulfate-polyacrylamide gels, separated electrophoretically, transferred to the polyvinylidene difluoride (PVDF) membrane, rinsed with Tris-buffered saline Tween (TBST), and then blocked in 5% nonfat milk buffer for 1 h at 37°C. Subsequently, the membranes were individually incubated overnight at 4°C and with the primary antibodies, including those against IκBα, p-IκBα, NF-κB p65, p-NF-κB p65, JNK, p-JNK, ERK1/2, p-ERK1/2, P38, p-P38, and β-actin. Then, the PVDF membranes were incubated with horseradish peroxidase-conjugated secondary antibodies for 2 h at room temperature. The images were captured by the ChemiDoc XRS instrument (Bio-Rad, Hercules, CA, United States). Using β-actin as the loading control, the densities of the protein bands were analyzed with ImageJ analysis software.

### Statistical Analysis

All data were analyzed using SPSS 20.0 software and displayed as the mean ± standard deviation (SD). Shapiro–Wilk test was used to examine whether the data were normally distributed. For the normally distributed data, if the data exhibited homogeneity of variance, statistical significance was carried out using one-way analysis of variance (ANOVA) followed by *LSD*′ post hoc test for comparisons among more than two groups; if the data did not exhibit homogeneity of variance, the differences were analyzed using Student’s *t*-test for comparisons between two groups. For the data without a normal distribution, the nonparametric Kruskal–Wallis test was applied. The *P* value less than 0.05 was regarded as statistically significant.

## Results

### Effects of Danhong Injection on Neurological Deficit Scores and Weight Loss Percentage in Rats

**FIGURE 1:**
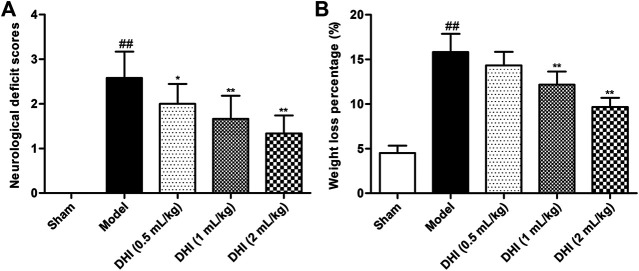
Effects of DHI on neurological deficit scores and weight loss percentage in CIRI rats. **(A)** The scores of the neurological deficit in all groups. **(B)** The body weight change of rats in all groups. Mean ± SD (*n* = 6). Asterisks denote the significant levels: ^##^ *P* < 0.01 vs. sham group and ^*^ *P* < 0.05 and ^**^ *P* < 0.01 vs. model group.

NDSs were evaluated by using the scoring criteria on a 5-point scale of Zea-Longa. As shown in Figure 1A, the scores of the sham group were zero, indicating no neurological deficits. After CIRI, the neurological deficit scores in the model group were significantly higher than those in the sham group (*P* < 0.01). Compared with the model group, the scores of DHI-treated groups (0.5, 1, and 2 ml/kg) were significantly lower than those of model group (*P* < 0.01 and *P* < 0.05), in a dose-dependent manner.

The decrease in body weight showed the same trend as neurological deficit scores. As shown in Figure 1B, the degree of body weight loss percentage in the model group was significantly higher than that in the sham group (*P* < 0.01). Compared with the model group, the DHI-treated groups (1 and 2 ml/kg) displayed obvious improvement in terms of weight loss percentage (*P* < 0.01). However, no significant difference was observed between the DHI-treated group (0.5 ml/kg) and the model group (*P* > 0.05).

### Effects of Danhong Injection on Cerebral Infarct Volume in Rats

**FIGURE 2:**
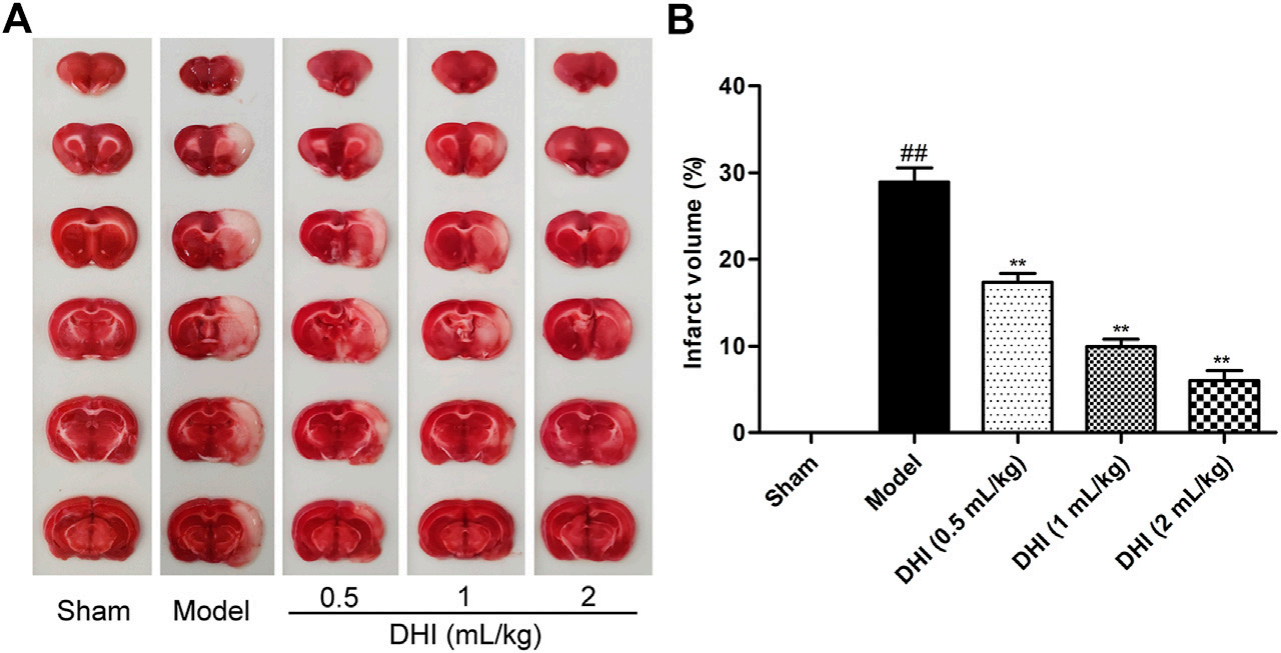
Effects of DHI on cerebral infarct volume in rats by TTC staining. **(A)** Representative images of TTC staining in all groups. **(B)** Results of cerebral infarct volumes in all groups. Mean ± SD (*n* = 6). Asterisks denote the significant levels: ^##^ *P* < 0.01 vs. sham group and ^**^ *P* < 0.01 vs. model group.

The cerebral infarction area was visualized using TTC staining. Representative stained images of TTC cerebral slices and quantified infarct volumes were shown in Figure 2. After CIRI, the infarct volume in the model group was significantly higher than that in the sham group (*P* < 0.01), indicating that a significant cerebral infarction appeared. Compared with the model group, the infarct volume was dose-dependently decreased in the DHI-treated groups at doses of 0.5 ml/kg, 1 ml/kg, and 2 ml/kg (*P* < 0.01). Data also showed that rats treated with DHI decreased the infarct volume from approximately 28% in the model group to approximately 17%, 10 %, and 6% in the DHI-treated groups at doses of 0.5 ml/kg, 1 ml/kg, and 2 ml/kg, respectively.

### Effects of Danhong Injection on Histopathological Changes in the Cerebral Cortex

**FIGURE 3:**
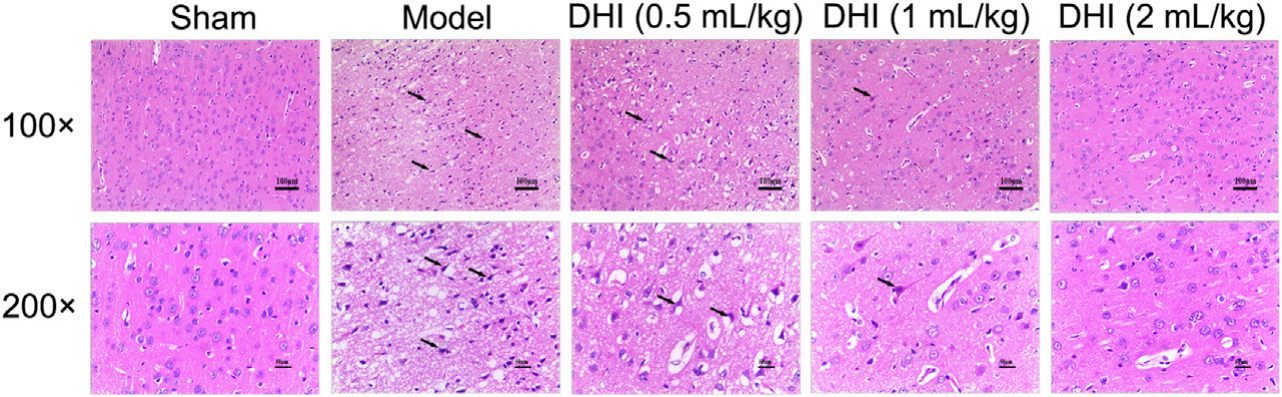
Effects of DHI on the pathological changes in the penumbra of ischemic ipsilateral parietal cortex at 24 h after CIRI in rats. Representative images of H&E staining at 100× and 200× magnification, and the black arrows indicate necrotic cells.

As shown in Figure 3, no histopathological abnormalities were observed in the sham group, and images showed clear cell contours, integrated tissue structures, obvious nucleoli, and no intracellular edema in the cerebral cortex. After CIRI, many cells in the penumbra of ischemic ipsilateral parietal cortex area showed shrinkage with deeply stained and condensed nuclei (karyopyknosis) that were surrounded by triangular pyknosis swollen cellular. Meanwhile, edematous cells, loose cytoplasm, severe cell deformation, and necrosis were also observed. In comparison with the model group, the abovementioned pathological abnormalities were all alleviated in different degrees after DHI administration in the three groups.

### Effects of Danhong Injection on mRNA Expressions of TNF-α, IL-1β, ICAM-1, COX-2, and iNOS in Ischemic Brains After Cerebral Ischemia-Reperfusion Injury

**FIGURE 4:**
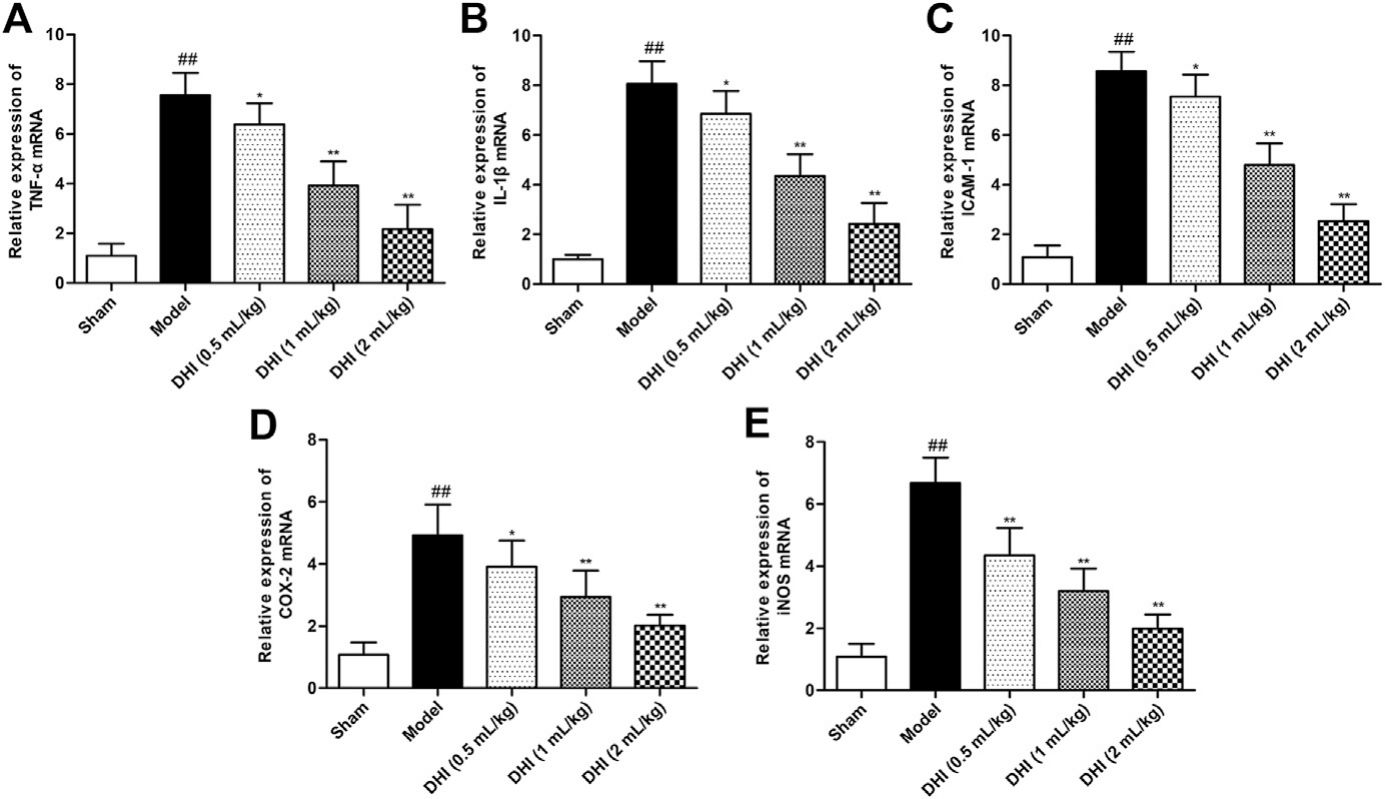
Effects of DHI on mRNA expressions of TNF-α, IL-1β, ICAM-1, COX-2, and iNOS in ischemic brains after CIRI. **(A)** TNF-α, **(B)** IL-1β, **(C)** ICAM-1, **(D)** COX-2, and **(E)** iNOS. Mean ± SD (*n* = 6). Asterisks denote the significant levels: ^##^ *P* < 0.01 vs. sham group and ^*^ *P* < 0.05 and ^**^ *P* < 0.01 vs. model group.

In order to evaluate the deeply effects of DHI on inflammatory responses, RT-PCR analysis was used to assess the mRNA expressions of TNF-α, IL-1β, ICAM-1, COX-2, and iNOS in ischemic brains. As shown in Figure 4, the mRNA expressions of TNF-α, IL-1β, ICAM-1, COX-2, and iNOS in ischemic brains were remarkably higher in the model group than those in the sham group (*P* < 0.01). In comparison with the model group, DHI-treated groups (0.5, 1, and 2 ml/kg) significantly decreased the mRNA expressions of TNF-α, IL-1β, ICAM-1, COX-2, and iNOS in ischemic brains (*P* < 0.01 and *P* < 0.05).

### Effects of Danhong Injection on Levels of TNF-α, IL-1β, and MCP-1 in Serum and MPO Activity of Ischemic Brains in Rats

**FIGURE 5:**
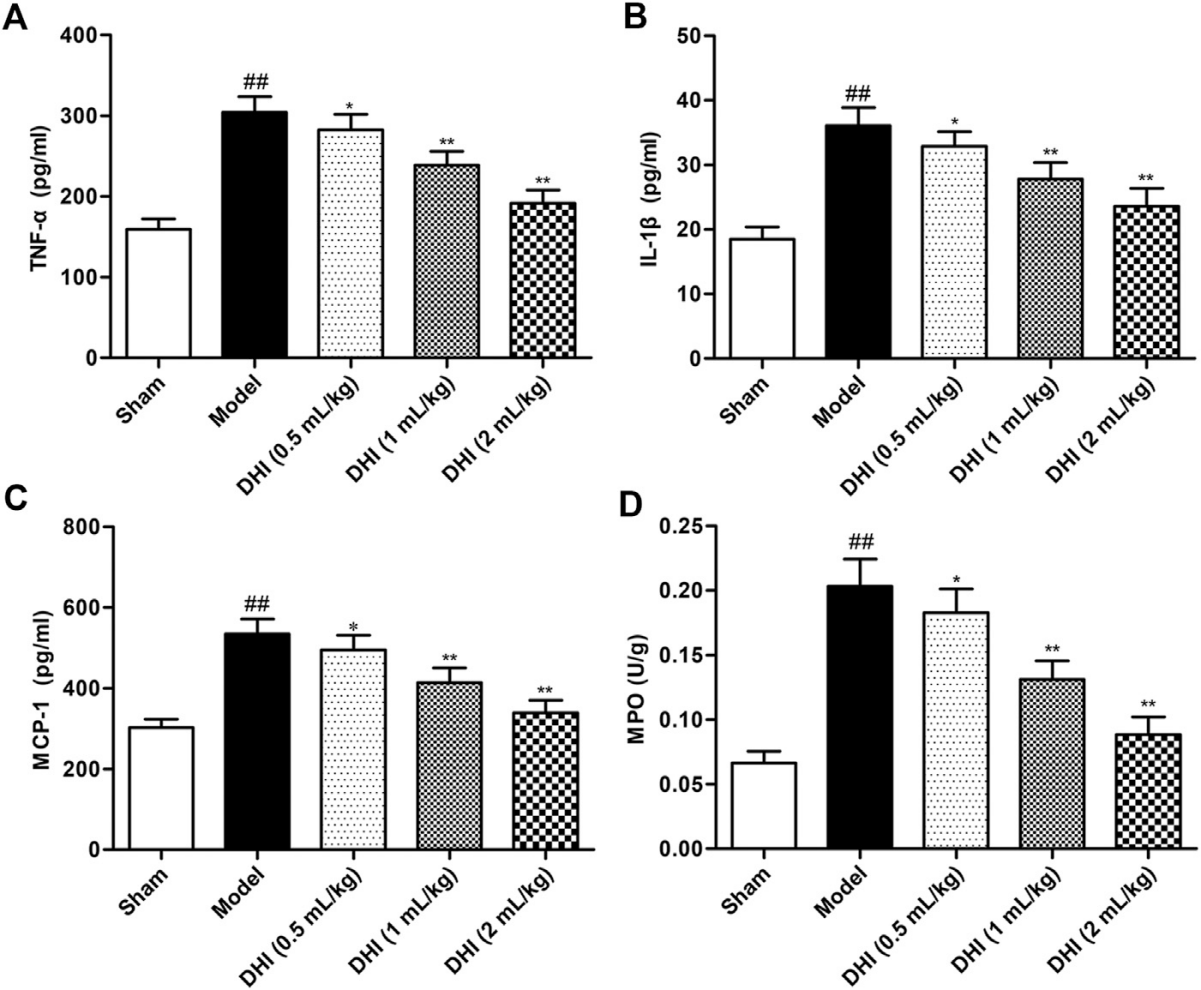
Effects of DHI on the levels of inflammatory cytokines in serum and MPO activity of ischemic brains in rats after CIRI. **(A)** TNF-α, **(B)** IL-1β, **(C)** MCP-1, and **(D)** MPO. Mean ± SD (*n* = 6). Asterisks denote the significant levels: ^##^ *P* < 0.01 vs. sham group and ^*^ *P* < 0.05 and ^**^ *P* < 0.01 vs. model group.

To explore the effects of DHI on the regulation of the inflammatory cytokine productions, the levels of TNF-α, IL-1β, and MCP-1 in serum were determined by ELISA kits. As shown in Figure 5, statistical analysis demonstrated that the levels of TNF-α, IL-1β, and MCP-1 were markedly increased in the model group relative to those in the sham group (all *P* < 0.01). In comparison with the levels in the model group, the levels of TNF-α, IL-1β, and MCP-1 in serum were significantly downregulated in the DHI-treated groups at doses of 0.5 ml/kg, 1 ml/kg, and 2 ml/kg (*P* < 0.01 and *P* < 0.05).

MPO activity was measured to evaluate the degree of leukocyte infiltration in ischemic brains. The experimental data demonstrated that MPO activity in ischemic brain tissues of the model group was significantly higher than that in the sham group (*P* < 0.01). However, this elevation exhibited a significant decrease in DHI-treated groups (0.5, 1, and 2 ml/kg).

### Effects of Danhong Injection on the Protein Expressions of Inflammatory Factors and Activation of Glial Cells in Ischemic Brains

**FIGURE 6:**
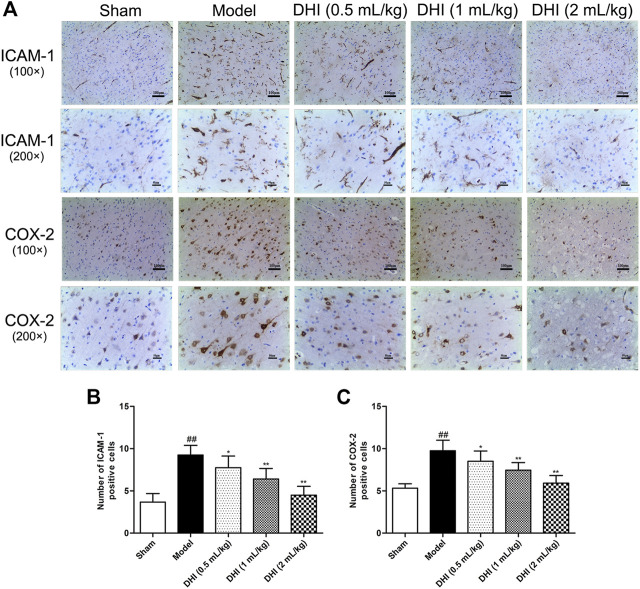
Effects of DHI on the protein expressions of ICAM-1 and COX-2 at 24 h after CIRI in rats. **(A)** Representative immunohistochemical images of ICAM-1 and COX-2, Iba-1, and GFAP in the penumbra of ischemic ipsilateral parietal cortex after CIRI in rats at 100× and 200× magnification. **(B, C)** Immunohistochemical analysis of ICAM-1 and COX-2 in the penumbra of ischemic ipsilateral parietal cortex after CIRI in rats. Mean ± SD (*n* = 6). Asterisks denote the significant levels: ^##^ *P* < 0.01 vs. sham group and ^*^ *P* < 0.05 and ^**^ *P* < 0.01 vs. model group.

To further explore pathological changes of neurogenic inflammation in each group, the expressions of inflammatory factors and activation of glial cells in ischemic brains were determined after CIRI. Immunohistochemical determination of ICAM-1 and COX-2 was performed to examine whether DHI regulated the protein expressions of the inflammatory factors. We measured the integrated optical density (IOD) of ICAM-1 and COX-2 in the cerebral cortex and calculated the mean values and SDs. As shown in Figure 6, immunohistochemical analysis showed that the protein expressions of ICAM-1 and COX-2 in the model group were significantly higher than those in the sham group (*P* < 0.01). In comparison with the model group, DHI-treated groups (0.5, 1, and 2 ml/kg) significantly downregulated the protein expressions of ICAM-1 and COX-2 (*P* < 0.01 and *P* < 0.05). These results are in accordance with the regulation effects of DHI on the mRNA levels of ICAM-1 and COX-2.

**FIGURE 7:**
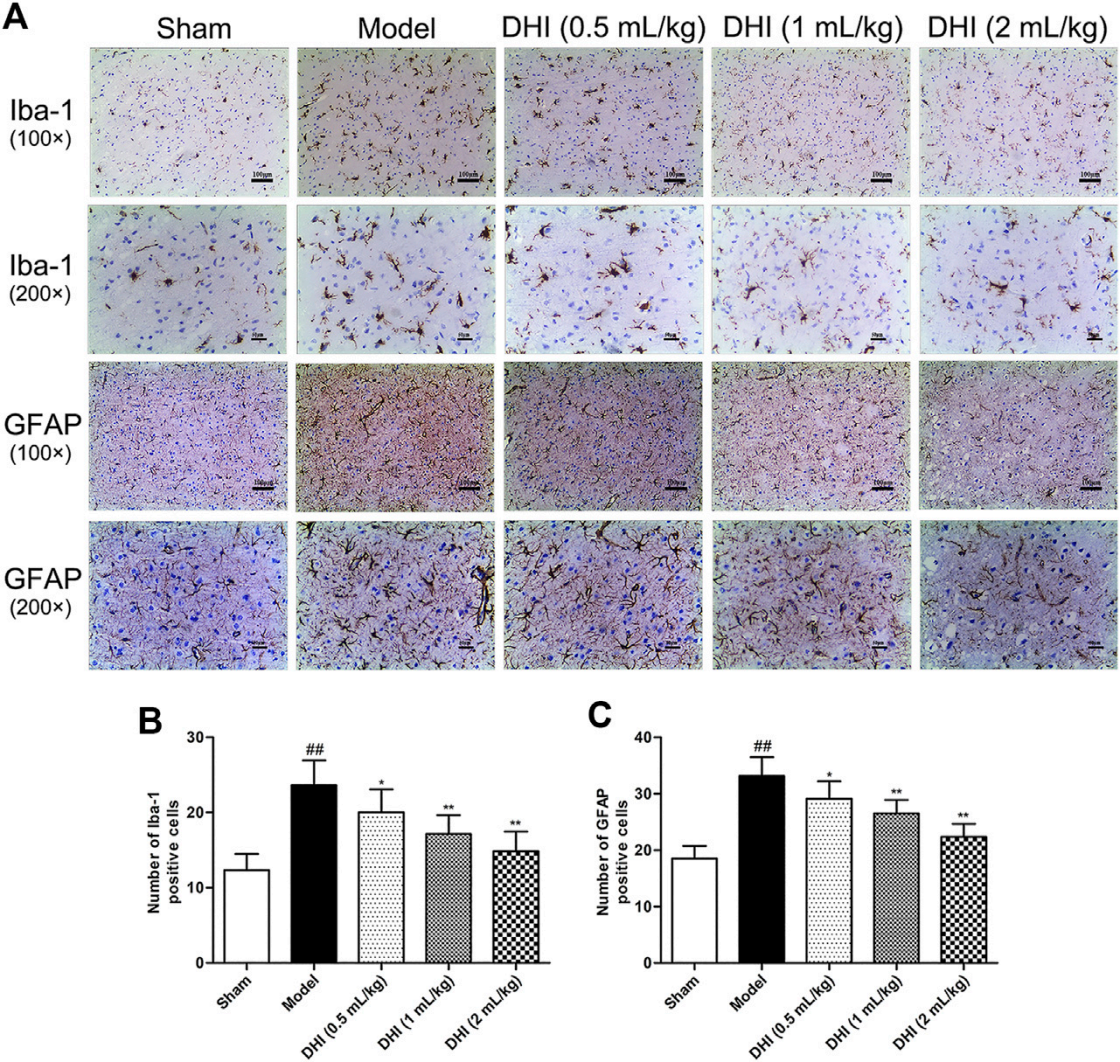
Effects of DHI on the protein expressions of Iba-1 and GFAP at 24 h after CIRI in rats. **(A)** Representative immunohistochemical images of Iba-1 and GFAP in the penumbra of ischemic ipsilateral parietal cortex after CIRI in rats at 100× and 200× magnification. **(B, C)** Immunohistochemical analysis of Iba-1 and GFAP in the penumbra of ischemic ipsilateral parietal cortex after CIRI in rats. Mean ± SD (*n* = 6). Asterisks denote the significant levels: ^##^ *P* < 0.01 vs. sham group and ^*^ *P* < 0.05 and ^**^ *P* < 0.01 vs. model group.

The activation of glial cells in CIRI involves microglia and astrocyte, so we also counted the numbers of Iba-1- and GFAP-positive cells in the cerebral cortex. As shown in Figure 7, the results showed that the rats in the model group exhibited more Iba-1- and GFAP-positive cells in the penumbra of ischemic ipsilateral parietal cortex regions than those in the sham group. However, in comparison with the model group, DHI-treated groups (0.5, 1, and 2 ml/kg) could significantly decrease the number of Iba-1- and GFAP-positive cells (*P* < 0.01 and *P* < 0.05).

### Effects of Danhong Injection on the NF‐κB and MAPK Signaling Pathways

**FIGURE 8:**
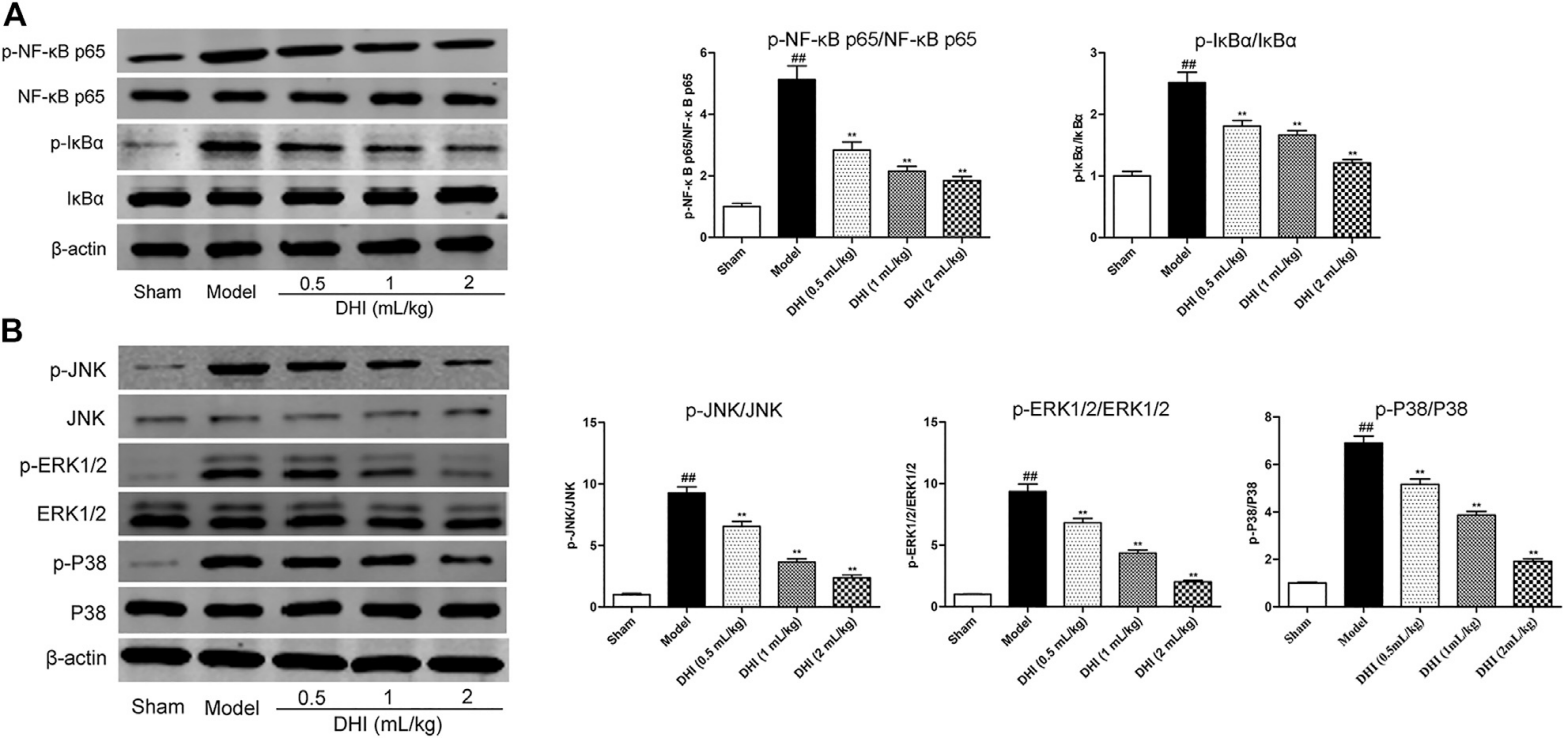
Effects of DHI on the protein expressions of the phosphorylated and nonphosphorylated forms of key effectors related to the NF-κB and MAPK signaling pathways by western blot analysis at 24 h after CIRI. **(A)** The representative bands of western blot in protein expressions of the phosphorylated and nonphosphorylated forms of NF-κB p65, IκBα, and β-actin in ischemic brain tissues; the quantitative analysis of protein expressions of p-NF-κB p65/NF-κB p65 and p-IκBα/IκBα, respectively. **(B)** The representative bands of western blot in protein expressions of the phosphorylated and nonphosphorylated forms of JNK, ERK1/2, P38, and β-actin in ischemic brain tissues; the quantitative analysis of protein expressions of p-JNK/JNK, p-ERK1/2/ERK1/2, and p-P38/P38, respectively. Mean ± SD (*n* = 3). Asterisks denote the significant levels: ^##^ *P* < 0.01 vs. sham group and ^*^ *P* < 0.05 and ^**^ *P* < 0.01 vs. model group.

Western blot assay was used to explore the protein expressions of the phosphorylated and the nonphosphorylated forms of NF-κB p65 and its inhibitor (IκBα) in ischemic brain tissues. As shown in Figure 8A, the ratio of p-NF-κB p65/NF-κB p65 and p-IκBα/IκBα in the model group was markedly higher than those in the sham group (*P* < 0.01). In comparison with the model group, DHI-treated groups (0.5, 1, and 2 ml/kg) significantly inhibited the protein expressions of the phosphorylated forms of NF-κB p65 and IκBα (*P* < 0.01 and *P* < 0.05).

In addition, we also examined the protein expressions of the phosphorylated and the nonphosphorylated forms of JNK, ERK1/2, and P38 in ischemic brain tissues by western blot assay. As shown in Figure 8B, the results showed a significant increase in the ratio of p-JNK/JNK, p-ERK1/2/ERK1/2, and p-P38/P38 in the model group compared with the sham group (*P* < 0.01). In comparison with the model group, DHI-treated groups (0.5, 1, and 2 ml/kg) could significantly inhibit the phosphorylation levels of JNK, ERK1/2, and P38 in ischemic brain tissues, respectively (*P* < 0.01 and *P* < 0.05), indicating that DHI may regulate the phosphorylation levels of JNK, ERK1/2, and P38 to produce a cerebral anti-neuroinflammatory effect.

## Discussion

In this study, a rat MCAO/R model was used to explore the therapeutic effects of DHI on early inflammatory injury of cerebral ischemia. The intraluminal suture method is the most commonly used animal model to study the neuroprotective effect of drugs in ischemic stroke (Yao et al., 2014). The results initially revealed that DHI had protective and therapeutic effects against CIRI in MCAO/R model rats. The evidence was that DHI could improve neurological function, decrease infarct volume, ameliorate pathological morphology, and inhibit inflammatory response in the areas of cerebral infarct. The pharmacological effect of DHI occurs in a dose-dependent manner. Moreover, we also explored the possible mechanism for the anti-neuroinflammatory effects of DHI on ischemic stroke.

Neuroinflammation plays a vital role in the pathophysiology of CIRI. Some pro-inflammatory substances, such as cytokines, chemokines, and adhesion molecules, are produced in the stroke model, and their inhibition or deficiency has been related to the reduction of CIRI (Ahmad and Graham, 2010; Kong et al., 2014). TNF-α and IL-1β, as the pro-inflammatory cytokines released under different stimulation of many cell types, play a critical role in glia activation and infiltration of inflammatory cells, further aggravating brain edema and enlarging the damage to ischemia nerve cell injury after CIRI (Huang et al., 2015). Studies found that TNF-α promotes the production of oxygen free radicals and the release of excitatory amino acid and also induces microglia and astrocyte to express cytotoxic iNOS during the inflammatory procedure (Wang Y. et al., 2013). IL-1β is the main form of IL-s in brain tissue and is elevated after CIRI, and it can not only cooperate with other cytokines to promote B and T cells activation but also regulate the production of TNF-α and participate in the pathological processes of other inflammatory cytokines (Yang et al., 2016). Taken together, TNF-α, IL-1β, and iNOS play important roles in the pathogenesis of CIRI. Consistent with the results of previous studies (Lee et al., 2016), the levels of TNF-α, IL-1β, and iNOS in our experiment were markedly increased when stimulated by CIRI. In comparison with the model group, DHI (0.5, 1, and 2 ml/kg) treatment dose-dependently decreased the TNF-α, IL-1β, and iNOS levels. These changes demonstrated that DHI might exert the protective effects by inhibiting the levels of inflammation cytokines after CIRI.

Cytokines and chemokines play an important role in the transcellular migration of leukocytes such as neutrophils, lymphocytes, and monocytes/macrophages (Shichita et al., 2012). Chemokines (such as MCP-1), as a family of small cytokines, could be used as chemoattractants to guide cell migration, promote leukocyte infiltration into ischemic brain tissue, and enhance inflammatory response during cerebral ischemia-reperfusion (Wang Y. et al., 2013). Previous studies found that the level of MCP-1 in serum at 24 h after cerebral ischemia-reperfusion was 6 times higher than that in the sham operation group and gradually decreased over time (Zhang et al., 2016). In addition, MPO is a specific enzyme of neutrophil leukocyte, and its activity can reflect the degree of neutrophil leukocyte infiltration (Sadeghi et al., 2011). It has been found that infiltrating neutrophils can enhance the inflammatory response of brain, which further aggravates ischemic brain injury (Kong et al., 2014). In our experiment, the levels of MCP-1 and MPO activity were significantly higher in the model group than those in the sham group. DHI (0.5, 1, and 2 ml/kg) treatment markedly reversed these alterations, indicating that DHI exerts anti-inflammation properties by suppressing the overproduction of MCP-1 and MPO activity.

ICAM-1, as a cell-surface glycoprotein member of the immunoglobulin superfamily, participates in the trafficking and adhesion of leukocytes to activated ischemia endothelia in stroke, so ICAM-1-induced intervention has become a promising therapeutic strategy against stroke (Choi et al., 2011; Mizuma and Yenari, 2017). Under normal circumstance, the levels of ICAM-1 are low and are upregulated by cytokines after ischemia, which in turn causes inflammatory response and aggravates ischemic brain injury (Mizuma and Yenari, 2017). In addition, endothelial cells regulate the leukocyte recruitment by expressing inflammatory related factors such as ICAM-1, IL-6, and COX-2, so as to actively participate in inflammatory events (Wang et al., 2015). The brain damage in mice carrying COX-2 transgene was aggravated, which confirmed the harmful effect of COX-2 in the ischemic injury (Xiang et al., 2007). In accordance with these studies, our results showed that, after CIRI, the mRNA and protein expressions of ICAM-1 and COX-2 in the model group were significantly increased. The treatment of DHI (0.5, 1, and 2 ml/kg) could dose-dependently decrease the expressions of ICAM-1 and COX-2, so as to further alleviate CIRI-induced brain injury in rats.

Microglial cells are regarded as the macrophages of the CNS, which play crucial roles in recovery of neurons and the normal development of the brain (Kim et al., 2017). Also, microglia and astrocytes are activated during the cerebral ischemia, and they are important components of the cell and molecular pathways involved in the destructive responses induced by stroke (Yao et al., 2014). In our study, a significant increase of Iba-1- and GFAP-positive expressions was observed in ischemic cortex tissues after CIRI, which was dose-dependently reversed by the treatment with DHI (0.5, 1, and 2 ml/kg). These findings suggest that DHI inhibits microgliosis and astrocytosis, further explaining its protective properties against neuroinflammation.

To further explore the underlying anti-neuroinflammatory effect of DHI against CIRI, western blot analysis was used to detect the protein expressions of the phosphorylated and nonphosphorylated forms of IκBα, NF-κB p65, JNK, ERK1/2, and P38 MAPK, which are the key signaling molecules in the NF-κB and MAPK signaling pathway (Kim et al., 2017; Tan et al., 2019; Xie et al., 2019). NF-κB, as a key regulator of central inflammatory response, includes a total of five proteins that are mainly present in an inactivated state in the cytosol as either homo- or heterodimers, with p65/p50 subunit as the most common heterodimer (Rothschild et al., 2018). As an important subunit of NF-κB, p65 can modulate the binding of p50 and DNA to activate the downstream responses. All isoforms have a common Rel homology domain, which is responsible for DNA binding and binding to cytoplasmic inhibitory proteins, called inhibitor-κB (IκB) (Xiao et al., 2001). Under the condition of CIRI, NF-κB p65 is activated in endothelial cells, microglia, and infiltrating inflammatory cells and then directly or indirectly regulates some pro-inflammatory factors, including cytokines (such as TNF-α and IL-1β), chemokines (such as MCP-1), and adhesion molecules (such as ICAM-1), forming a complex inflammatory cascade network to initiate inflammatory responses (Jung et al., 2014; Xu et al., 2017). In general, the activation caused by IκB leads to the activation and translocation of NF-κB and the synthesis and release of inflammatory factors (Cheng et al., 2019). In the experiment, the significant decrease in protein expressions of the phosphorylation forms of NF-κB p65 and IκBα were measured in the model group in comparison with the sham group. This revealed that NF-κB signaling pathway was activated after CIRI. Western blot assay results showed DHI (0.5, 1, and 2 ml/kg) dose-dependently inhibited phosphorylation levels of NF-κB p65 and IκBα in ischemic brain tissues. These observations indicated that the anti-neuroinflammatory effects of DHI against CIRI was related to the inhibition of the NF-κB signaling pathway, which was consistent with a previous report that DHI played anti-inflammatory effects on the inflammation triggers, LPS, ox-LDL, or cholesterol crystal-induced phosphorylation of NF-κB p65 in vascular endothelial cells (Lyu et al., 2017).

Moreover, MAPKs are a series of serine/threonine protein kinases that participate in the cerebral ischemic cascade and regulate CIRI-induced neuroinflammation, apoptosis, and autophagy (Wang P.-R. et al., 2013; Jiang et al., 2014). MAPK signaling pathways, as the target and mediator of CIRI, has been received increasing attention, and its predominating effect is to activate three different cascades, including the JNK, ERK1/2, and P38 (Sun Y. et al., 2015). Consistent with the existing reports (Jiang et al., 2014; Xie et al., 2019), the protein expressions of the phosphorylation forms of JNK, ERK1/2, and P38 in the model group were markedly higher than those in the sham group, providing evidence for the activation of downstream protein phosphorylation of the MAPK signaling pathway. Interestingly, DHI (0.5, 1, and 2 ml/kg) significantly downregulated the protein expressions of the phosphorylated forms of JNK, ERK1/2, and P38 proteins in ischemic brains. These findings indicated that DHI ameliorated CIRI-induced the neuroinflammation potentially *via* modulating the activation of the MAPK signaling pathway. Finally, as the important regulator of ischemic cerebral vascular disease, the NF-κB and MAPK signaling pathways have been reported to play a vital protective role in CIRI-induced inflammatory injury, raising the possibility that it might be a drug discovery target for stroke (Sun and Nan, 2016; Xie et al., 2019). These findings, together with our results, indicated that DHI exerted the anti-neuroinflammatory effects through the suppression of the activation of the NF-κB and MAPK signaling pathway.

## Conclusion

In summary, the results of our study revealed that DHI had a protective effect against CIRI in MCAO/R model rats in terms of the improvement of neurological function, the reduction of cerebral infarct volume, and the alleviation of pathological morphology in the cerebral cortex. Moreover, we also demonstrated that DHI could inhibit the production of inflammation-related molecules (TNF-α, IL-1β, ICAM-1, COX-2, and iNOS), decrease the levels of chemokine (MCP-1), reduce the neutrophil infiltration (MPO), and suppress the activation of glial cells (Iba-1 and GFAP). More importantly, DHI could attenuate neuroinflammatory injury in ischemic brains by modulating the NF-κB and MAPK signaling pathways. The pharmacological effect of DHI occurs in a dose-dependent manner. Taken together, these results may improve our understanding of the neuroinflammatory protection potential of DHI for treating CIRI in rats through suppressing the production of pro-inflammatory substances and the activation of the NF-κB and MAPK signaling pathways, which contribute to the amelioration of CNS damage.

## Data Availability

The raw data supporting the conclusions of this article will be made available by the authors, without undue reservation, to any qualified researcher.
