# Supplementary material for: Danhong Injection Attenuates Cerebral Ischemia-Reperfusion Injury in Rats Through the Suppression of the Neuroinflammation
Source: Front Pharmacol. 2021 Apr 13;12:561237. doi: 10.3389/fphar.2021.561237 (PMC8076794; doi:10.3389/fphar.2021.561237)
Supplement: Supplementary file 1 [file datasheet1.docx]

^
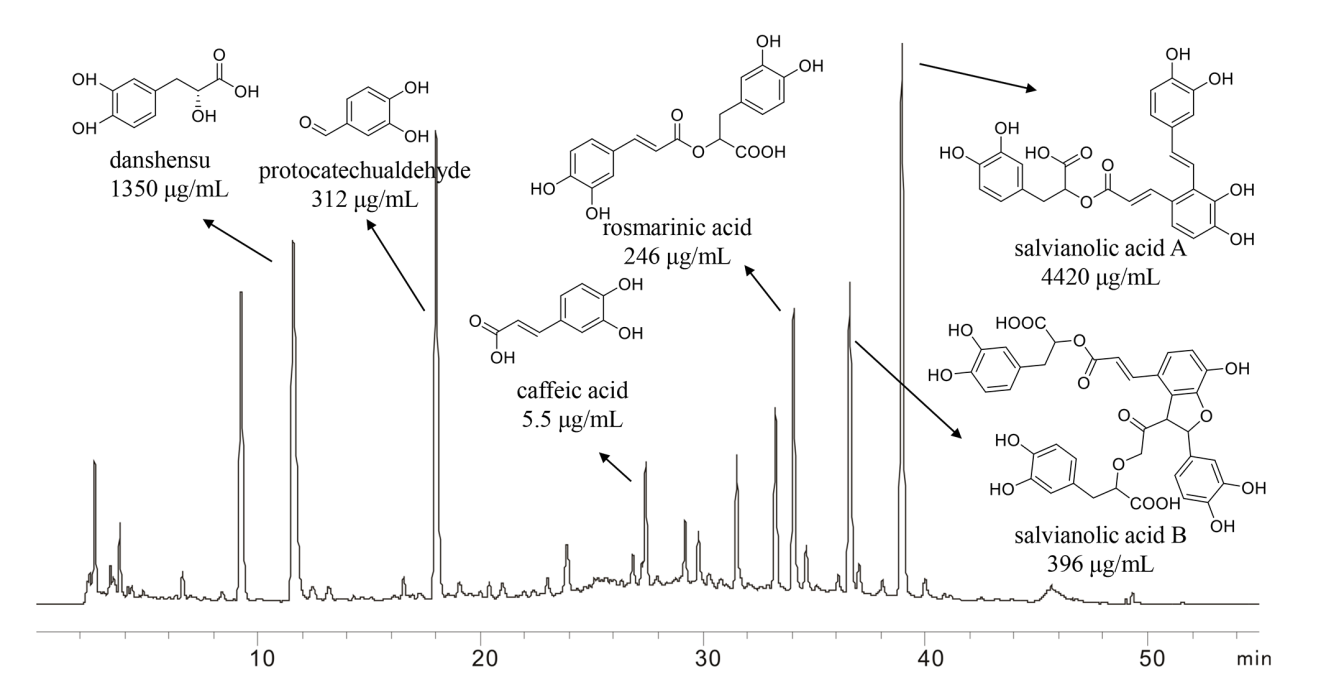
^^[[1]](#footnote-1)^**Figure S1**: Chromatogram of DHI by HPLC analysis at 280 nm. The chemical structures and relative contents of 6 major compounds were shown.


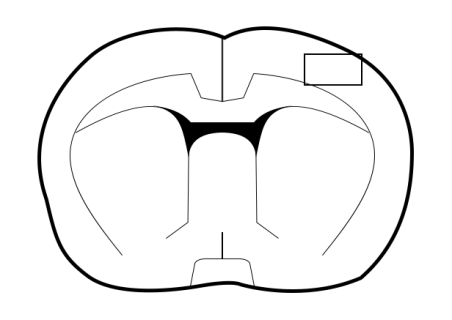


**Figure S2**: Schematic diagram of coronal brain section. The small rectangle in the penumbra of ischemic ipsilateral parietal cortex shows the histopathological selected area.

1. Data from unpublished results (Ling Zhang^#^, Yu Wang^#^, Chang Li, Chongyu Shao, Huifen Zhou, Jiehong Yang, Yu He^*^, Haitong Wan^*^. Contribution of Dan Hong Injection to the Myocardial Ischemia/Reperfusion Injury by improving cardiac mitochondrial function through Keap1/Nrf2/JNK pathway). The Danhong Injection used is the same batch as in this research. [↑](#footnote-ref-1)
